# Impact of pharmacist led mobile application on medication adherence and efficacy in chronic kidney disease

**DOI:** 10.1038/s41746-025-01742-8

**Published:** 2025-05-30

**Authors:** Shaza Gamal, Ahmad Mohamad Abbas Elseasi, Nirmeen Ahmed Sabry, Samar Farghali Farid

**Affiliations:** 1 Clinical Pharmacy Department, Faculty of Pharmacy, Cairo University, Cairo, Egypt; 2 National Institute of Urology and Nephrology, Cairo, Egypt

**Keywords:** Diseases, Health care, Medical research

## Abstract

Medication adherence is crucial for slowing chronic kidney disease (CKD) progression. A specially designed pharmacist-led mobile application, Kidney Health, was evaluated in a single-center, prospective, 3 months, randomized controlled trial (RCT) to assess its impact on medication adherence and efficacy in patients with CKD. 86 patients were randomly assigned to control group, who received standard of care, and app group, who in addition, had access to Kidney Health. The primary outcome was medication adherence measured by simplified medication adherence questionnaire (SMAQ). Other outcomes evaluated included kidney function, blood pressure, and random blood glucose (RBG). The app group showed a significant increase in adherent patients (*P* < 0.001) and decreased RBG (*P* = 0.047). Adherent patients increased (*P* = 0.002) and RBG decreased (*P* = 0.006) in app group compared to control group at the end of the study. Kidney Health has shown potential for encouraging patients with CKD to adhere to their medication and improving their clinical outcomes. Trial registration: clinicaltrials.gov with ID: NCT05168449, Date of registration: 12/2021 https://clinicaltrials.gov/study/NCT05168449.

## Introduction

Chronic kidney disease (CKD) is an irreversible and progressive disease with multiple possible complications if not properly managed^1^. CKD and cardiovascular disease-related deaths implicated to impaired kidney function caused 4.6% of global deaths, making CKD the 12th leading cause of death globally^2^. In Egypt, an estimated CKD prevalence of 7.32% equivalent to 7.3 million cases was reported in the Global Burden of Disease Collaborative Network in 2021^3^.

Medication adherence is critical in patients with CKD as it is crucial in slowing disease progression and improving health outcomes. Poor adherence in patients with CKD leads to a faster decrease in estimated glomerular filtration rate (eGFR) and is also associated with the occurrence of end stage renal disease (ESRD)^4^.

Patients with CKD receive an average of 12–19 prescribed medications^5^, inferring a high burden of pill intake with sometimes >20 pills/day^6^. Given the disease’s high pill burden and being life-long progressive disease, non-adherence in patients with CKD can reach 12%–53% in CKD stages 3–4^7–9^. There are few reports of adherence rates in Egyptian patients with CKD, an Egyptian study reported adherence of 50% in ESRD patients^10^. Nonadherence is associated with adverse outcomes and higher costs of care, increased morbidity, and mortality^6,11^. In renal transplant patients, clinical outcomes are impacted by adherence levels as in non-adherent patients compared to adherent patients, graft loss is seven times more likely^12,13^.

Many factors can help to improve medication adherence in patients with CKD. Pharmacists’ interventions were found to increase adherence, slow renal progression and improve other clinical outcomes in patients with CKD^14,15^. These outcomes can be attributed to the pharmacist’s vital role in disease management, optimizing drug therapy regimen, identifying barriers to non-adherence and patient education^14,16^.

In addition, patient education and knowledge about the disease and medications along with having positive attitudes regarding treatment can improve medication adherence^17^. Despite its importance, CKD awareness was found to be low among patients with CKD compared to awareness of other chronic diseases such as diabetes and hypertension^18^.

Also, new tools as mobile health (mHealth) can help reduce medication non-adherence. These tools proved crucial during COVID-19 pandemic as they had a significant impact on healthcare systems^19,20^. Newer digital adherence aids like mHealth, show potential in the management of chronic diseases, promotion of behavior change among patients, improving health outcomes, and reducing health care costs^21,22^.

mHealth in the form of mobile applications (mobile apps), can be used to improve medication adherence, monitor^23^ and have real time communication with patients in any setting^24^. According to the Egyptian Ministry of Communications and Information Technology, there were 41.42 million mobile internet subscribers in Egypt in 2020^25^, and this number rose to about 70 million in 2023^26^. Thus these interventions present an extensive opportunity for more accessible patient education and behavioral change tool.

There are a few studies with different approaches to assess the impact of mobile apps on CKD, but no randomized controlled trials assessing medication adherence. The aim of this study is to test the hypothesis that a pharmacist-led mobile application can improve adherence and efficacy of medication in patients with chronic kidney disease over a 3 months follow-up period as well as when compared to the standard of care.

## Results

### Baseline characteristics

**Fig. 1:**
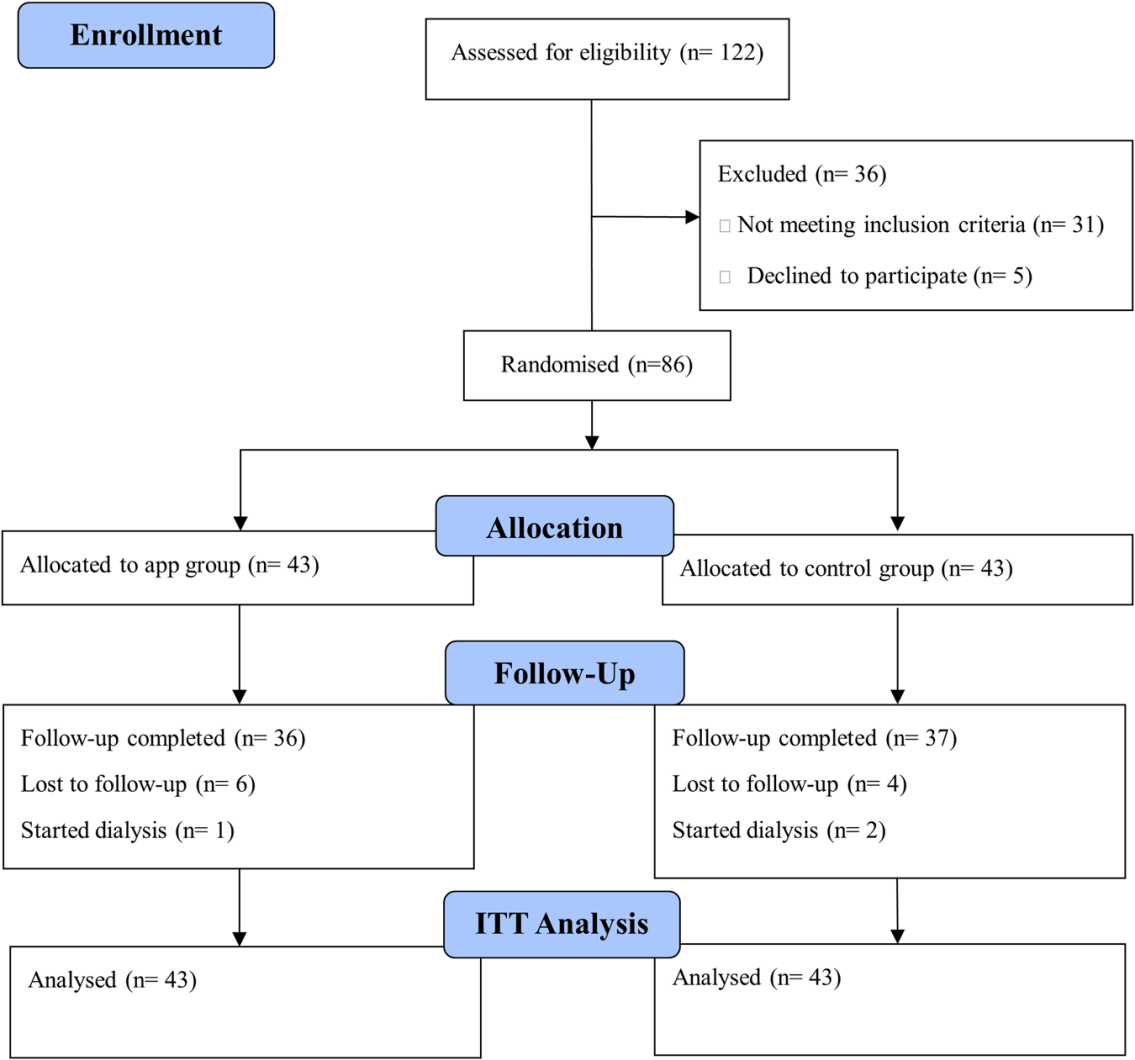
Consort patient’s flow chart. 86 patients were randomly assigned to the app group or control group, of whom 73 completed the study and 13 were either lost to follow-up or started dialysis. An Intention to Treat (ITT) analysis was performed and included all 86 enrolled patients.

A total of 122 patients with CKD were screened and 86 were randomized (43 in each group) during the period between November 2022 and September 2023 (Fig. 1). Of the 86 patients 10 patients were lost to follow-up and 3 were excluded as they started dialysis. All patients were included and an intention to treat analysis was carried out. Completers’ analysis results are provided in the supplementary Table 1 to Table 5.

The two groups showed homogeneity with no statistically significant differences in all baseline variables. Demographic and clinical characteristics are summarized in Table 1.

**Table 1:** Patients’ demographics, medications, and disease history expressed as mean (SD) or number of patients (percentage)

Variable	App Group (*n* = 43)	Control Group (*n* = 43)	*P* value*
Age, Years, Mean (SD)	36.91(12.90)	38.77(14.21)	0.527^a^
Gender, Male, *N* (%)	23 (53.50)	27 (62.80)	0.382^b^
Marital status, Married, *N* (%)	29 (67.40)	25 (58.10)	0.348^b^
Educational level, *N* (%)			
• Middle or lower	5 (11.60)	6 (14.00)	0.641^b^
• Secondary or equivalent	23 (53.50)	60.50)	
• University or higher	15 (34.90)	11 (25.60)	
Comorbidities, *N* (%)	36 (83.70)	40 (93.00)	0.313^c^
• Hypertension	26 (60.50)	28 (65.10)	0.655^b^
• Diabetes mellitus	8 (18.60)	11 (25.60)	0.436^b^
• Auto-immune disease	8 (18.60)	15 (34.90)	0.088^b^
• Dyslipidemia	10 (23.30)	11 (25.60)	0.802^b^
• Gout	15 (34.90)	14 (32.60)	0.820^b^
• Anemia	9 (20.90)	10 (23.30)	0.795^b^
Number of medications, Mean (SD)	7.74(2.80)	7.86(2.50)	0.839^a^
Medications, *N* (%)			
• Anti-hypertensives	38 (88.40)	35 (81.40)	0.526^b^
• Hypoglycemics	9 (20.90)	11 (25.60)	0.610^b^
• Immunosuppressants	33 (76.70)	33 (76.70)	1.00^b^
• Supplements	28 (65.10)	31 (72.10)	0.486^b^
• Lipid lowering agents	11 (25.60)	13 (30.20)	0.631^b^
• Uric acid lowering agents	15 (34.90)	16 (37.20)	0.688^b^
• Antiplatelets	8 (18.60)	8 (18.60)	1.00^b^
• Acid lowering agents	32 (74.40)	30 (69.80)	0.631^b^
Kidney disease stage, *N* (%)			
• 3A	27 (62.80)	25 (58.10)	0.905^b^
• 3B	6 (14.00)	(11.60)	
• 4	7 (16.30)	(20.90)	
• 5	3 (7.00)	4 (9.30)	
Period of CKD, Years (non-transplant patients), Mean (SD)	3.45 (4.96)	3.05 (3.91)	0.779^a^
Period of kidney transplant, Years, Mean (SD)	6.04 (2.29)	4.87 (3.40)	0.177^a^
History of Kidney transplant, *N* (%)	23 (53.50)	23 (53.50)	1.00^b^

*SD* standard deviation, *N* number of patients, *CKD* chronic kidney disease.

*: level of significance *P* < 0.05.

^a^Independent samples *t*-test.

^b^Chi square test.

^c^Fisher’s exact test.

### Between-group comparisons in app and control groups

Upon comparing the two groups after the introduction of the mobile app, results showed significant differences in SMAQ adherence and RBG. During follow-up 1 even though adherence percentages were higher in the app group, it did not reach statistical significance (*P* = 0.082). SMAQ adherence showed significant difference between both groups at follow-up 2 and 3 (*P* = 0.018 and 0.002 respectively) as shown in Table 2.

**Table 2:** Medication adherence and clinical outcomes in both groups overtime; expressed as mean (SD), median (IQR) or number of patients (percentage)

Outcome	Baseline	1 month	2 months	3 months	*P* value* (Within group)
SMAQ Overall Adherence, Adherent, *N* (%)					
App Group	4 (9.3)	20 (51.16)	27 (62.79)	**32 (74.42)**	**<0.001**^**a**^
Control Group	5 (12.2)	15 (34.90)	17 (39.53)	18 (41.86)	**0.019**^**a**^
* P* value* (Between groups)	0.738^b^	0.082^b^	**0.018**^**b**^	**0.002**^**b**^	-
eGFR (ml/min), Mean (SD)					
App Group	44.16 (15.74)	47.81 (15.82)	44.52 (17.79)	49.49 (18.5)	**<0.001**^**c**^
Control Group	41.37 (17.66)	41.72 (20.97)	44.33 (18.53)	43.44 (20.9)	0.305^c^
* P* value* (Between groups)	0.439^d^	0.091^d^	0.956^d^	0.127^d^	0.044**
SrCr, mg/dL Median (IQR)					
App Group	1.72 (1.45–2.30)	1.69 (1.33–2.40)	1.76 (1.46–2.54)	1.60 (1.21–2.23)	0.134^e^
Control Group	1.78 (1.57–2.9)	1.89 (1.52–3.17)	1.82 (1.52–2.80)	1.84 (1.52–2.97)	0.823^e^
*P* value* (Between groups)	0.292^f^	0.321^f^	0.577^f^	0.095^f^	–
Hemoglobin, g/dL, Mean (SD)					
App Group	11.81 (1.97)	11.90 (1.63)	12.11 (1.36)	11.72 (1.63)	0.350^c^
Control Group	11.97 (2.36)	12.09 (1.61)	11.99 (1.80)	11.80 (2.10)	0.755^c^
* P* value* (Between groups)	0.727^d^	0.650^d^	0.798^d^	0.843^d^	0.672**
Random blood glucose, mg/dL, Median (IQR)					
App Group	129 (105–150)	122.47 (106.20–134.14)	120.09 (103.60–131.14)	114 (103–123.04)	**0.047**^**e**^
Control Group	126.90 (106–155)	130.76 (116–145)	124.54 (111.94–133.65)	121.04 (114–139)	0.350^e^
* P* value* (Between groups)	0.966^f^	**0.042**^**f**^	0.260^f^	**0.006**^**f**^	-
Systolic blood pressure, Mean (SD)					
App Group	134.09 (21.44)	130.51 (16.52)	130.90 (14.35)	131.91 (17.76)	0.521^c^
Control Group	136.40 (20.25)	132.66 (11.47)	133.89 (12.87)	131.01 (14.24)	0.211^c^
* P* value* (Between groups)	0.609^d^	0.490^d^	0.318^d^	0.796^d^	0.704**
Diastolic blood pressure, Mean (SD)					
APP Group	83.44 (17.15)	81.37 (11.68)	81.30 (10.59)	81.61 (13.99)	0.736^c^
Control Group	87.12 (17.51)	82.42 (9.10)	82.75 (8.07)	80.55 (10.71)	**0.012**^**c**^
* P* value* (Between groups)	0.326^d^	0.628^d^	0.492^d^	0.695^d^	0.455**
Weight, Mean (SD)					
App Group	77.47 (20.21)	78.55 (19.32)	76.79 (15.24)	76.84 (20.17)	0.715^c^
Control Group	81.56 (23.77)	80.00 (15.32)	81.92 (12.10)	80.40 (15.09)	0.810^c^
* P* value* (Between groups)	0.390^d^	0.704^d^	0.096^d^	0.358^d^	0.723**

*eGFR* estimated glomerular filtration rate, *SrCr* Serum Creatinine, *IQR* Interquartile range.

*: level of significance *P* < 0.05, **: Interaction (Time × Group) *P* value, using mixed repeated measures ANOVA.

Bold values indicate *P* < 0.05.

^a^Cochrane Q test.

^b^Chi square test.

^c^One way repeated measures ANOVA.

^d^Independent samples *t*-test.

^e^Friedman test.

^f^Mann Whitney U test.

RBG measurements showed more prominent improvement in the app group compared to the control group over the three follow-ups (*P* = 0.042, 0.026, 0.006 respectively), that reached statistical significance at follow-ups 1 and 3 as shown in Table 2.

### Within group comparisons in app group

For within group analysis, results were significantly different for SMAQ adherence, eGFR, RBG and diastolic blood pressure. For the app group, SMAQ adherence increased significantly over time (*P* < 0.001). In pairwise comparison with Bonferroni correction, SMAQ adherence showed significant increase between the baseline and all the follow-up months (*P* < 0.001 for the three follow-ups), eGFR showed significant increase between follow-up 3 and both baseline and follow-up 2 (*P* = 0.006 and 0.002 respectively). RBG showed significant improvement within app group only, between baseline and follow-up 3 (*P* = 0.011).

### Within group comparisons in control group

While in the control group, pairwise comparison with Bonferroni correction showed significant difference in medication adherence between baseline, and both follow-up 2 and follow-up 3 (*P* = 0.08 and 0.003 respectively) and for eGFR, the control group did not show any significant increase from baseline over time (*P* = 0.305). For diastolic blood pressure, significant changes were found over time (*P* = 0.012), however pairwise comparison with Bonferroni correction showed no significant difference between any two time points.

### Kaplan-Meier analysis of medication adherence

**Fig. 2:**
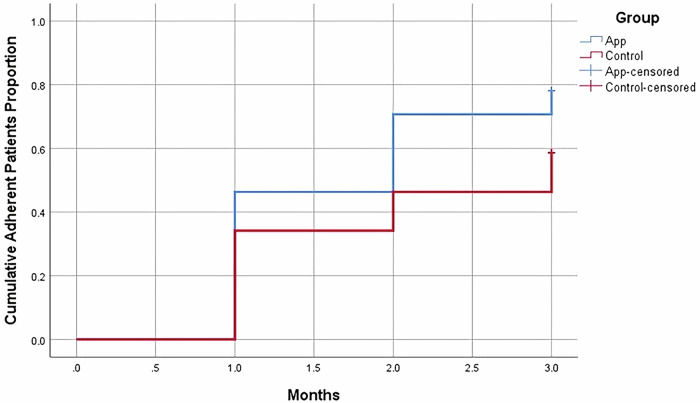
Kaplan-Meier representation of medication adherence in both groups overtime. The x-axis represents the follow-up months, and the y-axis represents the cumulative adherent patients’ proportion. Both app and control groups have shown an increase in cumulative adherent patients’ proportions over time. In the app and control groups, the median time for patients to become adherent was 2 and 3 months respectively. App group had a significantly higher cumulative adherent patients proportion compared to control group (*P* = 0.016).

Kaplan-Meier representation of the probability of becoming adherent in both groups overtime is shown in Fig. 2, where the event was patients becoming adherent and censored was patients remaining non-adherent throughout the follow-up period. There was a significant difference between both groups when comparing the time for patients to become adherent (*P* = 0.016) as shown in Table 3.

**Table 3:** The cumulative adherent patients’ proportions and median time for patients to become adherent in both groups

Group	Median time to becoming adherent (months)	Cumulative numbers and proportions of adherent patients at that time			Overall *P* value*
		1 month	2 months	3 months	
*App*	2	19 (0.473)	29 (0.717)	33 (0.795)	**0.016**^**a**^
*Control*	3	12 (0.292)	19 (0.460)	23 (0.569)	

*: level of significance *P* < 0.05.

Bold values indicate *P* value < 0.05.

^a^Log Rank (Cox Mantel) test.

### Kidney Health app usage and usability

Tables 4, 5 show app alarm responses and engagement of other app features. App alarm response showed significant increase over the follow-up period, where percentage of patients who responded to more than 25% of the alarms increased from 48.8% to 67.44% by the end of the study (*P* = 0.003), however upon pairwise comparison with Bonferroni correction, no significant difference was found between any of the follow-ups. ASUS score showed significant increase over the follow-up period, specifically between follow-up 3 and both follow-up 1 and 2 (*P* < 0.001and 0.022 respectively).

**Table 4:** App alarm response measured as percentage of alarms responded to and app usability and acceptability measured by ASUS overtime; expressed as number of patients and percentages

Outcome	1 month	2 months	3 months	*P* value* (within group)
*App alarm response N (%)*				
<25%	22 (51.2)	13 (30.2)	14 (32.56)	**0.003**^**a**^
25–50%	9 (20.9)	8 (18.6)	13 (30.23)	
50–75%	3 (7)	12 (27.9)	7 (16.28)	
≥75%	9 (20.9)	10 (23.3)	9 (20.93)	
*ASUS score, Mean (SD)*				
	68.43 (10.94)	70.94 (11.37)	76.02 (12.33)	**<0.001**^**b**^
*ASUS score Percentile, N (%)*				
< 68	24 (55.81)	18 (41.86)	10 (23.26)	**<0.001**^**c**^
≥68	19 (44.19)	25 (55.14)	33 (76.74)	

App alarm response is measured as percentage of total alarms the patient responded to, then percentages are divided into 4 groups (<25%, 25–50%, 50–75% and ≥75%).

*: level of significance *P* < 0.05.

Bold values indicate *P* value < 0.05.

^a^Friedman test.

^b^Repeated measures ANOVA.

^c^Cochran’s Q test.

**Table 5:** Other app features overall usage by patients; expressed as number of patients and percentages

App Features overall usage, *N* (%)	
*Reading the educational material*	35 (81.40)
*Educational quiz*	24 (55.81)
*Measurements recording*	13 (30.23)
*Side effects recording*	8 (18.60)
*Sending questions*	11 (25.6)

App features overall usage is defined as interacting with the feature either by answering a complete quiz, recording a measurement or side effect or sending a question and these entries getting recorded, at least once during the follow-up period. As for reading education materials, patients received daily notifications that when clicked would direct them to a different part in the educational materials section, and patients reporting at follow-up whether they have read these educational materials.

With regards to app features usage, of the 24 patients who completed an education quiz, 18 patients completed all the 3 provided quizzes. For measurements recording 6 patients made more than 2 entries with the maximum number of entries for 1 patient being 12. For side effects recording, the 8 patients made 1–2 entries. Only 2 patients of the 11 patients sent more than 2 questions.

There was a significant positive correlation between SMAQ adherence and app percentage alarm response (app measured adherence) in app group. Using Chi square association test, a correlation coefficient (Cramer’s V) of 0.286, 0.467 and 0.459 was estimated for each follow-up respectively. This correlation was significant for follow-up 2 and 3 (*P* = 0.027 and 0.035 respectively). There were no significant correlations found between SMAQ adherence and other app features.

## Discussion

Kidney Health app was introduced in the current single centered RCT to patients with CKD, stages 3–5 in the intervention group, providing them with features as medications recording, personalized daily alarms and response recording, educational material and notification, data logging of labs and symptoms, and sending questions to the pharmacist while the control group received the standard of care. Patients were followed up by the pharmacist for 3 months and their medication adherence, clinical outcomes as well as app usability were assessed. The app group showed significant improvement in medication adherence, probability of becoming adherent and in some clinical outcomes such as eGFR and RBG compared to the control group. To our knowledge this is the first RCT to assess the impact of pharmacist-led mobile app intervention on medication adherence and efficacy in patients with CKD stages 3–5.

mHealth interventions have been investigated previously in different chronic diseases showing efficacy and positive impact on medication adherence^27–30^. In a recent meta-analysis that included 14 RCTs, mobile applications were associated with significant improvement in medication adherence in adults with chronic diseases. The main features of the apps included in the analysis were documentation, medication reminder, data sharing, feedback message, clinical decision support and education^30^. In a 3 months study, Mira et al. assessed medication adherence, errors and clinical outcomes after introducing an mHealth intervention offering reminders and medication education to chronically ill elderly patients. Patients in the app obtained significantly higher adherence scores, fewer errors and missed doses compared to the control group, adding to the evidence of the positive impact of such interventions^27^.

Previous studies carried out in the CKD population included mHealth interventions such as lifestyle advice and real time communication with nurses, education by nephrologist and mHealth technologies as well as diet and exercise follow-up and education^31–33^. Two studies includes apps with multiple features of data recording, education, communication channel and only one of them included reminders^34,35^. All studies showed significant improvement in some of the measures outcomes. These outcomes included knowledge and self-management scores, survival analysis and some clinical outcomes like blood pressure and eGFR.

Our study is one of the few RCTs done to evaluate the impact of an mHealth intervention on pre-dialysis patients with CKD. Liu et al. and Tsai et al. had similar intervention and population; however, they were retrospective and prospective cohort studies respectively, with different outcomes measured not focusing on medication adherence and its importance in such population^31,34^. RCTs were carried out by Sarker et al., Li et al., and Maneesri et al. on similar population however, medication adherence was not reported, and there were conflicting clinical outcomes results. Moreover, the type of mHealth intervention widely varied mainly focusing on education and lifestyle management, besides none of them mentioned a pilot phase for the mHealth intervention used^32,33,35^. In the current study we attempted to overcome previous studies’ limitations by adding a pilot phase, measuring medication adherence by two methods and using a more adaptable mHealth intervention while implementing a pharmacist-led approach.

Pharmacist-led digital interventions have demonstrated noticeably positive impact on medication adherence and clinical outcomes in patients with complex diseases and medication management^29,36–38^. Our study involved a pharmacist-led intervention thus having an added advantage compared to self-management or other healthcare professional-led interventions as the pharmacist had direct and active involvement with the patients through training the patients, carrying out personalized monthly follow-up interviews, immediate response to app-submitted questions and careful assessment of medication adherence.

Pharmacist-led studies in transplant, hypertension and diabetes populations have shown similar benefits as the current study, nevertheless our study is the first to confirm these effects in CKD population through using robust adherence measures and an mHealth platform^29,37,38^. A study that investigated the efficacy of a pharmacist-led mobile application on medication adherence and diabetes-related outcomes among women with gestational diabetes and followed up for 12 months post-partum, found the involvement of a pharmacist, reduced patients’ medication suspicion and provided encouragement for raising their medication adherence^29^. The pharmacist-led intervention used, significantly enhanced medication adherence and improved glycemic control compared to usual care group. Another study that examined the efficacy of improving medication safety through a pharmacist-led medication therapy monitoring and management app in post-transplant patients showed that such intervention helped develop a partnership between patients and clinicians as well as mitigate patient safety issues^37^.

Having a pilot phase is another one of the current study’s strengths. In the study carried by Mira et al., the authors emphasized the importance of carrying out a pilot phase as it allowed the app to be more adaptable to patients’ requirements and more acceptable to them as well as to healthcare professionals^27^. Thus, the pilot phase allowed Kidney Health app design to be based on the views and preferences of the target patients and the experience of healthcare professional along with assessing the usability during the app development.

Medication adherence is crucial in this unique population. Medication adherence was measured using SMAQ questionnaire as well as app reminders response. SMAQ was chosen as it was found to be a valid and reliable scale as well as being simple, short with good psychometric properties^39,40^. The baseline SMAQ measured percentage adherent was low in both groups (9.3–12.2%), when compared to previously mentioned estimates of adherence in CKD patients^10^.

This study analyses demonstrated sustained improvements in adherence and some clinical outcomes that are not solely attributable to time effects or the standard care. We reported a significantly higher percentage of adherent patients in the app group compared to the control group. This is in concordance with previous results reported by Mira et al. and Khah et al., where mHealth interventions with either reminders or education materials in chronically ill elderly patients or hemodialysis patients respectively, were used and medication adherence significantly improved after 3 months follow-up^27,41^. In the current study the increase in adherence in the app group was evident from the first follow-up and significantly higher than the baseline throughout the study period. While, in the control group it became evident by follow-up 2 hence having a higher median time of becoming adherent as shown by the Kaplan-Meier analysis. Even though adherence was higher in the app group compared to the control throughout the study reaching significance at follow-up 2 and 3, both groups showed significant increase at the end of the study. Anglada et al. assessed medication adherence in a single arm study of chronically ill patients, where an app for medication recording and reminders was introduced, they discussed the Hawthorne effect where patients improve an aspect of their behavior as medication adherence in response to awareness of being observed^28^. This might be a possible explanation for the significant increase in adherence in the control group, especially that our study had a higher frequency, monthly, follow-up. Still, despite this possibility the app group have shown a significant improvement in medication adherence compared to the control group.

Adherence reported through the app, percentage of alarms responded to, has shown significant positive correlation starting from follow-up 2 with SMAQ measured adherence. This positive correlation shows promising potential, firstly as previous studies did not estimate adherence as previously mentioned and secondly supports using mHealth recorded responses as a measure for medication adherence especially that there is no gold standard for measuring adherence.

As for the clinical outcomes in this study, they were used as surrogates for assessing medication efficacy and by measuring and reporting the within group changes, we were able to detect significant improvements in the app group patients’ eGFR and RBG. Few studies reported the impact of an mHealth intervention on kidney functions, and they did not report the within group changes significance. Li et al., reported a significantly higher eGFR in the app group, who had access to app with diet and exercise recording and education, compared to control group at the end of the 3 months study^33^. While, similar to our study Sarker et al., showed no significant changes in kidney function between the app group, who received education by nephrologist along with mHealth technology interventions, and the control group at the end of the study^32^. In the current study, significant difference was found in the interaction of app × time for eGFR, also by the end of the study, the app group had higher eGFR compared to control but it did not reach significance between groups. One possible explanation for the improvement in eGFR in both groups, is that the significant increase in adherence within both groups in the current study might have helped in slowing the disease progression, as adherence correlates to slower disease progression^4^.

The observed changes in the eGFR and RBG in the Kidney Health app group show potential for the usefulness of such intervention in improving medication efficacy and clinical outcomes in patients with CKD, through increasing medication adherence, providing educational material and a communication channel between patients and Pharmacists. Furthermore, features such as recording lab results and providing trend charts are all potential factors that might have helped as well.

To our knowledge previous studies with CKD-oriented apps did not assess usability. Assessing app usability is important as it can dictate the adoption of such interventions, users’ willingness to use them, sustained engagement as well as their actual usefulness and clinical impact^42,43^. Therefore, usability measured in the current study reflected the patients’ experience and willingness to use the app. We used the 10 question Arabic SUS, which is considered a well-designed, balanced questionnaire consisting of 5 questions with positive statements and 5 questions with negative statements. It has become a common method for measuring the usability for different digital products including digital health applications^42^.

Kidney health app had an above-average ASUS mean score throughout the follow-up period as well as a significant increase in the percentile of patients giving the app a score greater than 68 at the end of the study. Even though our app showed good usability and an increasing interaction with the app alarms over the follow-up period, most patients responded to less than 25% of the alarms and app features requiring data entry as lab tests, side effects, and sending questions had relatively low interaction. There may be several reasons for these results, patients might have found it extra work, moreover, being used to other channels of contact with the healthcare professionals leading to low interaction^28^. Also some of the app features needed internet connection (e.g. sending a question or data recording), so they will not function properly if there is an internet connection problem.

Despite the strengths of our study being an RCT with a pilot phase, one of the study limitations was being single centered. Also, as it was not feasible to provide patients with smart phones, patients had to own a smartphone to be recruited so they were more likely to be willing to try and use this type of intervention. Furthermore, patients provided their own laboratory data, as the study was carried out in a private nephrology clinic, so it was not feasible to withdraw samples. Another limitation was that we could not be sure that patients took the medications, however a well-accepted and validated medication adherence measurement tool was used along with adherence reported through the app. Moreover, when evaluating the relationship between both measures of medication adherence in the app group, they showed good correlation.

In conclusion, our study demonstrated how a pharmacist-led mHealth intervention succeeded in encouraging patients to adhere to their medication regimens through its multiple integrated features, thus showing the potential of such adherence aids in practice and on wider scale to help patients with CKD to be more self-dependent with the guidance of healthcare professionals such as the pharmacists to improve their medication adherence and efficacy.

## Methods

### Study design

This study was a single-center, prospective, open-label, parallel group (1:1), randomized controlled trial (RCT). It was conducted in a private nephrology clinic, Cairo, Egypt. The study involved the introduction of a mobile application to patients with CKD to improve their adherence to the prescribed medications. Recruited patients were randomized into two groups using Random Allocation Software with equal block size of 10. The control group was provided with standard of care, defined as routine monthly visits to the clinic’s physician, while the intervention group was provided in addition to the standard of care, with access to a specially designed mobile application after simple training on app usage. Both groups were followed up and interviewed by the pharmacist monthly for a period of 3 months.

### Participants

Patients were screened for recruitment from November 2022 to September 2023. Patients were selected to participate in this study based on the inclusion criteria of age between 18–65 years, eGFR < 60 ml/min/1.73 m^2^, literate, ownership of android smartphone compatible with the application, polypharmacy (defined as 5 or more medications daily)^44^, with exclusion criteria of being on dialysis or pregnancy.

The sample size was calculated using G*power 3.1.9.7 software, based on a previous study by Gomis et al.^45^, with effect size of 0.86 in medication adherence as the primary outcome. A total of 52 patients with 1:1 allocation would be required to achieve 80% power at a two-sided significance level of 0.05. Assuming 15% dropout rate, a sample size of 60 patients was needed.

### Outcome measures and data collection

The primary outcome measured was medication adherence and secondary outcomes included control of kidney function (eGFR), blood pressure, hemoglobin, weight and random blood glucose (RBG). Also, application usability was measured.

Demographic data was collected at baseline. Both groups were evaluated at baseline and monthly, at follow-up 1, 2 and 3, for medication adherence, clinical outcomes and application usability. Medication adherence, for all CKD and non-CKD medications in patients’ clinic records, was assessed using Arabic version of Simplified Medication Adherence Questionnaire (SMAQ). The SMAQ consists of 6 questions assessing medication adherence: 1) Do you ever forget to take your medicine?, 2. Are you careless at times about taking your medicine?, 3) Sometimes if you feel worse, do you stop taking your medicines?, 4) Last week, how often have you not taken your medicine?, 5) Did you not take any of your medicine over the past weekend?, 6) Over the past 1 month, how many days have you not taken any medicine at all? A patient is considered non-adherent when there is a positive response to any of the first four qualitative questions, a response of more than two doses missed over the past week to question 5, or a response of not taking any prescribed medication over 2 days during the past month to question 6^39,40^.

eGFR was calculated using the Chronic Kidney Disease Epidemiology Collaboration (CKD-EPI) formula. Also, serum creatinine (SrCr), blood pressure, hemoglobin level, weight and RBG data were collected. Glycated hemoglobin (HbA_1C_) was assessed at baseline and after 3 months that is at follow-up 3. In addition to the previously mentioned measures, the application usability and acceptability by the intervention group was assessed monthly using the 10-item Arabic System Usability Scale (ASUS) for mobile applications being integrated in the application and application usage was also assessed. SUS has 10 questions, each question with a Likert scale ranging from strongly agree to strongly disagree with total score ranging from 0-100 where a score <68 is considered below average and ≥ 68 above average^46–48^.

### Intervention

The intervention included 4 stages: Mobile application design, development, validation and implementation. The mobile application named Kidney Health was designed by the investigators of this study to have the features of patient profile, personalized medication reminders, symptoms and measurements recording, educational material and sending questions. The education material and content has been prepared and revised by the investigators, among them a nephrology consultant.

The main app features included: The patient can log in to his account, record general information in the profile, record any measurement from the list of measurements provided (e.g. SrCr, random blood glucose, blood pressure), choose symptoms or side effects, browse through educational material, answer educational quizzes and receive the correct answers, as well as sending questions to the pharmacist and receiving responses to them. Also, receiving educational as well as app use motivational notifications along with generation of trend charts for measurements were part of the service.

All data entered by patients were recorded in the app and app website. The medication reminders included a “Yes or No” for the patient to indicate whether the medication was taken or not with time of response being recorded in the app and the app website. The application was in Arabic language and was made available on google play store.

**Fig. 3:**
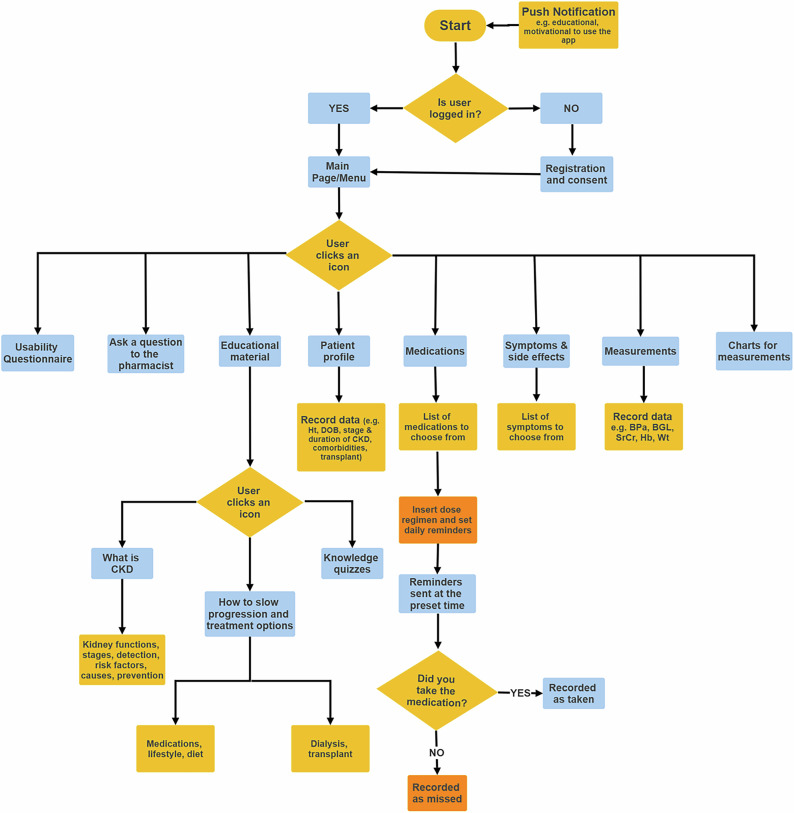
Design flow chart for Kidney Health app. The design of Kidney Health consisted of login page directing the user to the main menu page to enter the username and password. The main menu showcases the different app features including (profile, medications and alarms, symptoms and side effects, measurements and charts, educational material, sending a question to the pharmacist and the usability questionnaire). Patients recorded information such as kidney disease stage and duration, comorbidities, height, educational level and date of birth on the profile page. Patients can select their medications from a provided list and record them and set daily reminders. Patients can also record side effects, measurements such as blood pressure and serum creatinine, create trend charts for these measurements as well as send questions to and receive answers from the pharmacist. Educational material section included various pages with information and advice related to CKD as well as educational quizzes.

The mobile application was developed by “The Center of advanced software and biomedical engineering consultants - Faculty of Engineering- Cairo University” based on the app design provided to them by the investigators (Fig. 3). The application was developed using Android Studio and Flutter software, with the coding language “JAVA”. The database management program was developed using MYSQL. The patients used the android mobile application, and the investigator accessed the patients’ data through an application website. The application website connected all data recorded to the patient’s username and allowed data download in the form of excel files.

The validation stage included beta testing by the development team for any bugs followed by validation by the investigators including 19 users (9 patients with CKD, 4 non-CKD subjects, 4 pharmacists, 2 nephrologists) to test features of the app and provide feedback for any more errors or bugs and modifications to be done in user interface or language. They also provided feedback on the two questionnaires used in the study, the A-SUS for mobile applications and the Arabic version of SMAQ.

**Fig. 4:**
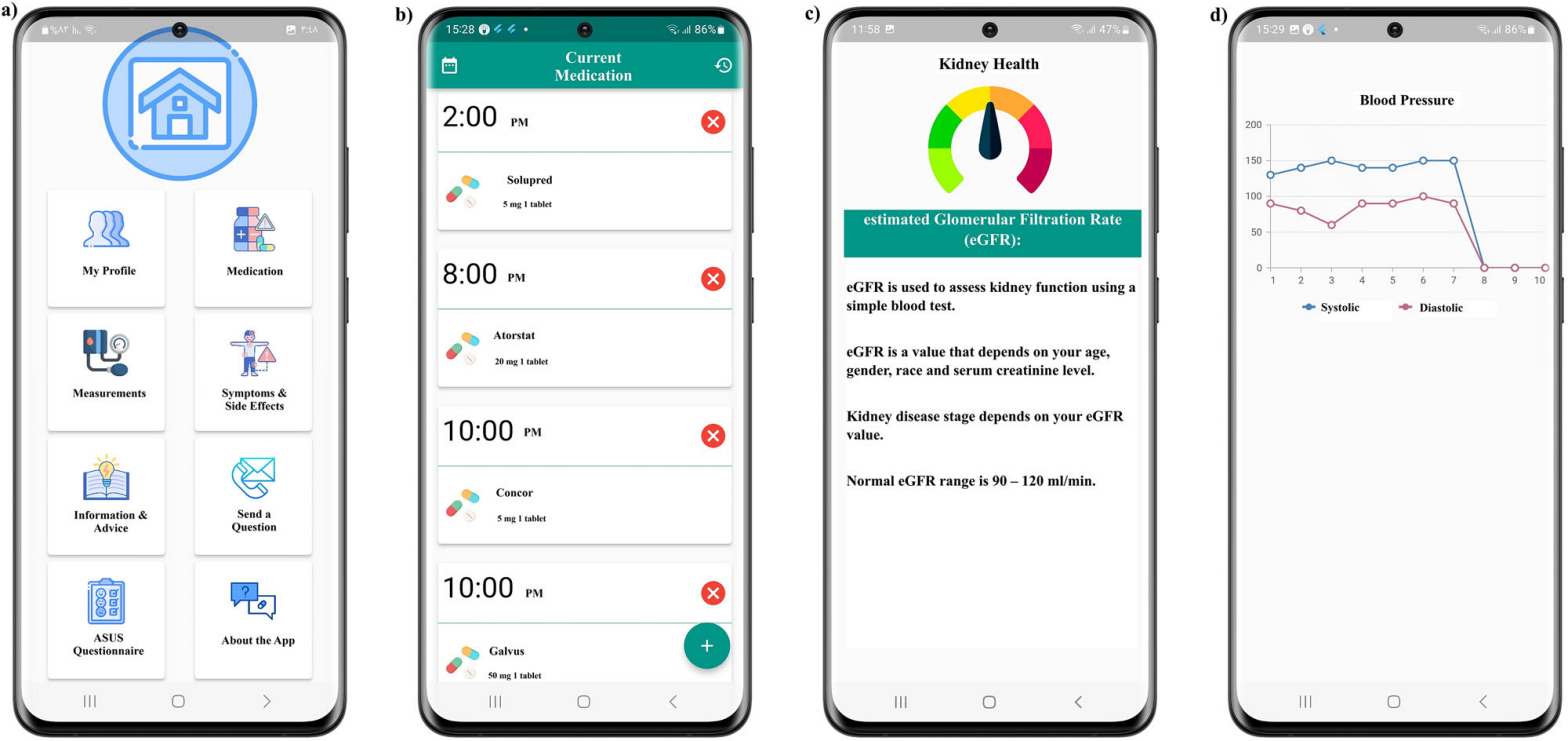
Screenshots of some of the Kidney Health app features. Main menu page with the main features of the app (**a**), recorded current medications and alarms with time and doses (**b**), an example of educational material page stating the definition and importance of estimated glomerular filtration rate (**c**) and an example of a trend chart that shows most recent 10 measurements, created from previously recorded systolic and diastolic blood pressure (**d**).

Kidney health app validation process resulted in the discovery of some bugs that were fixed during beta testing. These included alarms not ringing properly, log-in issues and notifications not being sent. After fixing all the bugs such as reminders not ringing, notifications not showing and recorded data not getting sent to the website, the app got validated by testing on 20 users who used it for a week and then gave feedback on their experience. Based on this feedback, some improvements were made such as changing the alarm setting clock format to be easier, two-step alarm deletion was added so alarms would not be deleted accidentally, displaying trend graphs for each measurement parameter separately and changes were made to the backgrounds and fonts colors to offer a clearer interface. Examples of some of the app features are shown in Fig. 4 and Supplementary Movies 1 and 2.

The language of the app, ASUS and SMAQ questionnaires also were reviewed during this phase. For the app there were some typing and punctuation corrections. Regarding the adopted questionnaires, as they have been already validated, only minor changes were made to some wording in ASUS, so it was retested, and reliability analysis was done with a resulting Cronbach’s alpha of 0.754.

The implementation phase consisted of the patients’ visits to the clinic, patients’ interviews by the pharmacist and app implementation and usage by the patients during the follow-up period. The patients’ journey started with their clinic visits at baseline and each month where all patients received usual care in the clinic. The usual care consisted of the clinic nurses recording the patients’ labs, measuring their blood pressure, weight and random blood glucose at the beginning of their visit and having their routine meeting with the physicians. In addition, at the baseline visit all patients had the recruitment interview with the pharmacist for about 20–30 min, where patients in both groups were introduced to the study, data was collected, medications recorded on the clinics’ system were reconciled and baseline medication adherence was assessed by the pharmacist. In addition, the app group had Kidney Health downloaded on their smartphones, app settings were adjusted, and each patient was given a username and password to access the app with. Patients were trained by the pharmacist to use the app during the recruitment interview. They were trained to use the app by recording and responding to the medication alarms, reading education material, recording any measurements or symptoms as well as sending any questions or concerns to the pharmacist through the app and were asked to use the app on daily basis during the 3 months follow-up period. While at follow-up visits, instead of the recruitment interview, patients had a follow-up interview with the pharmacist that included medication reconciliation and medication adherence assessment for both groups, in addition app usage and usability were assessed for the app group.

### Ethical approval

Informed consent was obtained from all patients before entering the study. Ethical approval was obtained from Cairo University research ethics committee, Approval: CL 2871. The study is registered on clinicaltrials.gov with ID: NCT05168449. Confidentiality of the patient’s data was protected by using username and password, generated for each patient account and data sent from patients’ accounts can be accessed from the backend of the server, through a password protected application website.

### Statistical analysis

Statistical analysis was done using IBM SPSS Statistics, version 26. For descriptive statistics, categorical variables are expressed as the number of patients and their percentages, variables are expressed as mean and standard deviation or as median and interquartile range (IQR) based on normality. Intention to treat analysis was carried out where multiple imputation was used to account for missing data and the pooled values of 5 iteration were used. Nominal outcomes were compared between the two groups using Chi squared test or Fisher’s exact. Continuous outcomes were assessed using independent *t*-test or Mann Whitney U between the two groups depending on normality. For within group assessment, nominal outcomes were compared using Cochran’s Q test followed by pairwise analysis, while continuous outcomes were compared using repeated measures ANOVA or Friedman test followed by pairwise analysis with Bonferroni correction. Correlation was measured using Chi square association test.

## Supplementary information

Supplementary Information

Supplementary Movie

Supplementary Movie

## Data Availability

The datasets analyzed in the current study are not publicly available due to patient privacy purposes but are available upon reasonable request to the corresponding author Shaza Gamal (shaza.gamal@pharma.cu.edu.eg).

## Code availability

The code can be made available upon reasonable request to the corresponding author Shaza Gamal (shaza.gamal@pharma.cu.edu.eg).
