# Supplementary material for: Impact of pharmacist led mobile application on medication adherence and efficacy in chronic kidney disease
Source: NPJ Digit Med. 2025 May 30;8:325. doi: 10.1038/s41746-025-01742-8 (PMC12125339; doi:10.1038/s41746-025-01742-8)
Supplement: Supplementary file 1 — Supplementary Information [file 41746_2025_1742_MOESM1_ESM.pdf]

Supplementary Table 1. Patients' demographics, medications, and disease history expressed as mean (SD) or number of patients (percentage) (Completers only)

Variable	App Group N=36	Control Group N=37	P value*
Age, Years, Mean (SD)	35.47 (12.59)	39.1 6 (14.43)	0.256 [a]
Gender, Male, N (%)	19 (52.78)	22 (59.50)	0.565 [b]
Marital status, Married, N (%)	23 (63.90)	21 (56.80)	0.373 [b]
Educational level, N (%)			
• Middle or lower	4 (11.10)	6 (16.2)	0.737 [b]
• Secondary or equivalent	21 (58.30)	22 (59.2)	
• University or higher	11 (30.60)	9 (24.3)	
Comorbidities, N (%)	30 (83.30)	35 (94.60)	0.124 [c]
• Hypertension	21 (58.30)	24 (64.90)	0.566 [b]
• Diabetes mellitus	6 (16.70)	9 (24.30)	0.418 [b]
• Auto-immune disease	8 (22.20)	12 (32.40)	0.328 [b]
• Dyslipidemia	7 (19.40)	11 (29.70)	0.308 [b]
• Gout	11 (33.30)	13 (35.10)	0.871 [b]
• Anemia	7 (19.40)	9 (24.30)	0.614 [b]
Number of medications, Mean (SD)	7.42 (2.761)	7.86 (2.529)	0.305 [a]
Medications, N (%)			
• Anti-hypertensives	32 (88.90)	31 (83.80)	0.526 [b]
• Hypoglycemics	7 (19.40)	9 (24.30)	0.614 [b]
• Immunosuppressants	30 (83.30)	27 (73.00)	0.285 [b]
• Supplements	21 (58.30)	27 (73.00)	0.188 [b]
• Lipid lowering agents	8 (22.20)	12 (32.40)	0.328 [b]
• Uric acid lowering agents	12 (33.30)	14 (37.80)	0.688 [b]
• Antiplatelets	6 (16.70)	7 (18.90)	0.801 [b]
• Acid lowering agents	26 (72.20)	26 (70.30)	0.854 [b]
Kidney disease stage, N (%)			
• 3A	24 (66.67)	56.76	0.853 [b]
• 3B	4 (11.11)	13.51	
• 4	6 (16.67)	21.62	
• 5	2 (5.56)	8.10	
Period of CKD, Years (non-transplant patients), Mean (SD)	4.07 (5.63)	3.17 (4.12)	0.60 [a]
Period of kidney transplant, Years, Mean (SD)	4.74 (2.90)	4.31(3.12)	0.073 [a]
History of Kidney transplant, N (%)	21 (58.30)	19 (51.4)	0.549 [b]

*: level of significance $P < 0.05$

a: Independent samples t-test

b: Chi square test

c: Fisher's exact test

SD: Standard deviation; N: Number of patients; CKD: Chronic kidney disease.

Supplementary Table 2. Medication adherence and clinical outcomes in both groups overtime; expressed as mean (SD), median (IQR) or number of patients (percentage) (Completers only)

Outcome	Baseline	1 month	2 months	3 months	P value* (Within group)
SMAQ Overall Adherence, Adherent, N (%)					
App Group	3 (8.3)	18 (50)	22 (62.9)	28 (77.8)	**<0.001**[a]
Control Group	5 (13.5)	12 (33.3)	13 (36.1)	15 (40.5)	**0.019** [a]
P value (Between groups)	0.71[b]	0.151 [b]	**0.034** [b]	**<0.001** [b]	-
eGFR (ml/min), Mean (SD)					
App Group	45.47 (15.74)	48.94 (15.82)	44.48 (17.79)	49.97 (18.5)	**<0.001**[c]
Control Group	41.57 (17.66)	42.66 (20.97)	44.61 (18.53)	42.92 (20.9)	0.934 [c]
P value (Between groups)	0.323 [d]	0.184 [d]	0.979 [d]	0.132 [d]	0.408**
SrCr, mg/dL, Median (IQR)					
App Group	1.70 (1.45-2.29)	1.64 (1.3-1.88)	1.71 (1.49-2.42)	1.19 (1.61 – 2.08)	**0.043** [e]
Control Group	1.78 (1.57-2.81)	1.81 (1.53-3.03)	1.77 (1.58-2.57)	1.82 (1.52-2.82)	0.952[e]
P value (Between groups)	0.265[f]	0.127 [f]	0.582 [f]	0.075 [f]	-
Hemoglobin, g/dL, Mean (SD)					
App Group	11.86 (1.97)	11.83 (1.77)	12.15 (1.38)	11.74 (1.75)	0.251[c]
Control Group	11.79 (2.24)	12.31 (1.84)	12.06 (2.31)	11.95 (2.35)	0.810 [c]
P value (Between groups)	0.894 [d]	0.377 [d]	0.880 [d]	0.691 [d]	0.856**
Random blood glucose, mg/dL, Median (IQR)					

App Group	130 (103.25-152.25)	111 (102.75-134.75)	109 (98-135)	110.5 (101.25-118.75)	0.213 [e]
Control Group	123.5 (103.75-153)	129 (107.25-163)	113 (99.75-159.5)	120 (113.25-140.5)	0.539 [e]
P value (Between groups)	0.817 [f]	0.064 [f]	0.345 [f]	**0.005** [f]	-
Systolic blood pressure, Mean (SD)					
App Group	135.31 (21.63)	130.71 (19.07)	130.19 (17.96)	131.31 (18.76)	0.99 [c]
Control Group	135.16 (20.52)	132.17 (12.76)	134.73 (17.66)	130.97 (15.29)	0.277 [c]
P value (Between groups)	0.977 [d]	0.727 [d]	0.380 [d]	0.934 [d]	0.892**
Diastolic blood pressure, Mean (SD)					
APP Group	84.39 (18.31)	81.29 (12.37)	80.74 (13.34)	80.17 (12.29)	0.967 [c]
Control Group	84.54 (16.04)	81.34 (9.84)	83.50 (10.77)	80.46 (11.48)	**0.045** [c]
P value (Between groups)	0.970 [d]	0.985 [d]	0.437 [d]	0.917 [d]	0.842**
Weight, Mean (SD)					
App Group	77.19 (20.81)	78.17 (23.19)	75.43 (18.98)	77.54 (21.66)	0.556 [c]
Control Group	80.29 (15.71)	81.04 (16.19)	84.68 (15.75)	80.54 (16.25)	**0.011** [c]
P value (Between groups)	0.475 [d]	0.587 [d]	0.070 [d]	0.507 [d]	**0.038****

a: Cochrane Q test
b: Chi square test
c: One-way repeated measures ANOVA
d: Independent samples t-test
e: Friedman test
f: Mann Whitney U test
*: level of significance P <0.05
**: Interaction (Time x Group) P value, using mixed repeated measures ANOVA
eGFR: estimated glomerular filtration rate; SrCr: Serum Creatinine; IQR: Interquartile range

Supplementary Table 3. The cumulative adherent patients' proportions and median time for patients to become adherent in both groups. (Completers only)

Group	Median time to becoming adherent (months)	Cumulative numbers and proportions of adherent patients at that time			Overall P value*
		1 month	2 months	3 months	
**App**	2	18 (0.486)	26 (0.703)	29 (0.784)	**0.014**[a]
**Control**	3	10 (0.286)	15 (0.429)	18 (0.514)	

a: Log Rank (Cox Mantel) test
*: level of significance P <0.05

Supplementary Table 4. App alarm response measured as percentage of alarms responded to and app usability and acceptability measured by ASUS overtime; expressed as number of patients and percentages. (Completers only)

Outcome	1 month	2 months	3 months	P value* (within group)
**App alarm response N (%)**				
**<25 %**	17	13	14	0.076 [a]
**25 – 50 %**	7	5	10	
**50 – 75 %**	5	10	7	
**≥ 75%**	7	8	5	
**ASUS score, Mean (SD)**				
	68.47 (11.82)	70.90 (12.35)	75.9 (13.41)	**0.004**[b]
**ASUS score Percentile, N (%)**				
**< 68**	21 (58.30)	17 (47.22)	10 (27.70)	**0.001**[c]
**≥ 68**	15 (41.70)	19 (52.78)	26 (72.30)	

a: Friedman test
b: Repeated measures ANOVA
c: Cochran's Q test
*: level of significance P <0.05
App alarm response is measured as percentage of total alarms the patient responded to, then percentages are divided into 4 groups (< 25%, 25-50%, 50-75% and ≥ 75%).

Supplementary Table 5. Other app features overall usage by patients; expressed as number of patients and percentages

**App Features overall usage, N (%)**	
**Reading the educational material**	31 (86.10)
**Educational quiz**	18 (50.00)
**Measurements recording**	11 (30.56)
**Side effects recording**	8 (22.22)
**Sending questions**	6 (16.67)

Movies legends

Supplementary Movie 1: Different features of Kidney Health app. The steps of logging in and adding new medication and alarm are shown. Followed by recording new measurements such as serum creatinine and random blood glucose with a trend chart representation. Symptoms and side effects page is shown and some examples of educational material pages. An example of entering a question is shown as well.

Supplementary Movie 2: Kidney Health alarm response and other features. The alarm and how to respond to it is shown. Also, list of previously recorded alarm responses and an answer to a sent question is shown.

CONSORT 2010 checklist of information to include when reporting a randomised trial*

Section/Topic	Item No	Checklist item	Reported on page No
**Title and abstract**			
	1a	Identification as a randomised trial in the title	Not added due to word count
	1b	Structured summary of trial design, methods, results, and conclusions (for specific guidance see CONSORT for abstracts)	2
**Introduction**			
Background and objectives	2a	Scientific background and explanation of rationale	3-4
	2b	Specific objectives or hypotheses	4
**Methods**			
Trial design	3a	Description of trial design (such as parallel, factorial) including allocation ratio	10
	3b	Important changes to methods after trial commencement (such as eligibility criteria), with reasons	N/A
Participants	4a	Eligibility criteria for participants	10
	4b	Settings and locations where the data were collected	10
Interventions	5	The interventions for each group with sufficient details to allow replication, including how and when they were actually administered	12-14
Outcomes	6a	Completely defined pre-specified primary and secondary outcome measures, including how and when they were assessed	11

	6b	Any changes to trial outcomes after the trial commenced, with reasons	N/A
Sample size	7a	How sample size was determined	11
	7b	When applicable, explanation of any interim analyses and stopping guidelines	N/A
Randomisation:			
Sequence generation	8a	Method used to generate the random allocation sequence	10
	8b	Type of randomisation; details of any restriction (such as blocking and block size)	10
Allocation concealment mechanism	9	Mechanism used to implement the random allocation sequence (such as sequentially numbered containers), describing any steps taken to conceal the sequence until interventions were assigned	10
Implementation	10	Who generated the random allocation sequence, who enrolled participants, and who assigned participants to interventions	13
Blinding	11a	If done, who was blinded after assignment to interventions (for example, participants, care providers, those assessing outcomes) and how	N/A
	11b	If relevant, description of the similarity of interventions	N/A
Statistical methods	12a	Statistical methods used to compare groups for primary and secondary outcomes	14
	12b	Methods for additional analyses, such as subgroup analyses and adjusted analyses	N/A
**Results**			
Participant flow (a diagram is strongly recommended)	13a	For each group, the numbers of participants who were randomly assigned, received intended treatment, and were analysed for the primary outcome	19

	13b	For each group, losses and exclusions after randomisation, together with reasons	19
Recruitment	14a	Dates defining the periods of recruitment and follow-up	4
	14b	Why the trial ended or was stopped	N/A
Baseline data	15	A table showing baseline demographic and clinical characteristics for each group	23
Numbers analysed	16	For each group, number of participants (denominator) included in each analysis and whether the analysis was by original assigned groups	19
Outcomes and estimation	17a	For each primary and secondary outcome, results for each group, and the estimated effect size and its precision (such as 95% confidence interval)	24-27
	17b	For binary outcomes, presentation of both absolute and relative effect sizes is recommended	24-27
Ancillary analyses	18	Results of any other analyses performed, including subgroup analyses and adjusted analyses, distinguishing pre-specified from exploratory	N/A
Harms	19	All important harms or unintended effects in each group (for specific guidance see CONSORT for harms)	N/A
**Discussion**			
Limitations	20	Trial limitations, addressing sources of potential bias, imprecision, and, if relevant, multiplicity of analyses	9-10
Generalisability	21	Generalisability (external validity, applicability) of the trial findings	10
Interpretation	22	Interpretation consistent with results, balancing benefits and harms, and considering other relevant evidence	7-10
**Other information**			

Registration	23	Registration number and name of trial registry	14
Protocol	24	Where the full trial protocol can be accessed, if available	N/A
Funding	25	Sources of funding and other support (such as supply of drugs), role of funders	15

Citation: Schulz KF, Altman DG, Moher D, for the CONSORT Group. CONSORT 2010 Statement: updated guidelines for reporting parallel group randomised trials. BMC Medicine. 2010;8:18.
© 2010 Schulz et al. This is an Open Access article distributed under the terms of the Creative Commons Attribution License (http://creativecommons.org/licenses/by/2.0), which permits unrestricted use, distribution, and reproduction in any medium, provided the original work is properly cited.

*We strongly recommend reading this statement in conjunction with the CONSORT 2010 Explanation and Elaboration for important clarifications on all the items. If relevant, we also recommend reading CONSORT extensions for cluster randomised trials, non-inferiority and equivalence trials, non-pharmacological treatments, herbal interventions, and pragmatic trials. Additional extensions are forthcoming: for those and for up-to-date references relevant to this checklist, see www.consort-statement.org.
